# Who Discovered Anæsthesia?

**Published:** 1859-10

**Authors:** 


### *Who Discovered Anæsthesia?*

Nineteen years ago this summer, Dr. L. P. Brockett, a physician now, but then a student in Hartford, Connecticut, having recently had a molar tooth extracted which gave him great pain, was talking with the dentist on various subjects, when the conversation turned on the intoxicating influences of nitrous oxide gas. The dentist remarked, "that he believed that a man might be made so drunk by this gas, or some similar agent, that dental or other operations might be performed upon him without any sensation of pain on the part of the patient." This conversation occurred in August, 1840, and the man who uttered the startling and entirely novel proposition, was Horace Wells.

Four years passed by, and in the same city a traveling lecturer (Colton by name) administered to several persons the "laughing gas," amongst others, to a certain dentist. One of the party, while under its influenee, received a severe hurt, but did not give any evidences of pain, when the dentist remarkad to his neighbor (Mr. David Clarke), that he believed "that a man, by taking that gas, could have a tooth extracted or a limb amputated, and not feel the pain." This was on the 10th of September, 1844; and the great idea was again distinctly stated by the same Horace Wells.

On the morning of the 11th of September (the day after his lecture), Mr. Colton was requested by a gentleman to go with him to a dentist of Hartford, Dr. J. M. Riggs, and carry some "nitrous oxide." This person sat down in the operating chair, took the bag of gas, and inhaled it until he became insensible, and Dr. Riggs extracted one of his largest teeth. On coming to his senses, he crien out "it did not hurt me more than the prick of a pin; it is the greatest discovery ever made." On that day the great idea became an embodied fact, and the discoverer proved in his own person the truth of his theory, for the man was Horace Wells.

From that time his restless, excitable spirit knew no peace. Day and night he talked of it, experimented with it, and studied its effects and modes of preparation. In a few months the truth was verified by many successful experiments. Doctors and professors, bishops, members of Congress, and many citizens of Hartford and the vicinity united with one accord to declare, from personal experience, their perfect faith in the new discovery. Not only in tooth drawing, but in large surgical operations was the experiment tested. The thigh was amputated, tumors removed, cancers dissected out of the human body without pain: and for twenty-two months no other man opened his mouth, made an experiment or published a fact with regard to the great discovery about to bestow its priceles blessing on suffering humanity, save the one to whom we owe it—HORACE WELLS.

This ardent, zealous seeker after truth, often injudicious and extravagant, but ever frank and guileless, had a quondam student (now friend,) who lived in Boston. His name was W. T. G. Morton. To him he applies for assistance, so that his discovery may be brought before the notice of the great men of the metropolis. Morton gave him the opportunity of using the nitrous ozide in the presence of the medical class of Harvard university. The tooth was extracted, but the patient screamed; and although he afterwards declared that he did not feel the pain, the students hissed the trembling adventurer (the unknown dentist) from the hall—and back to his home, heart broken, friendless, but not desparing came HORACE WELLS.

The tale is almost told. Morton sees his chance. Wells had proved that *sulphuric ether* has the wonderful power; and fearing to repeat an experiment which had just failed, he determines to try the other. He seeks for an influential friend, and finds him in *Professor* Charles T. Jackson (God save the mark)! and on the 30th of September 1846, twenty-two months after Dr. Wells proved the fact on himself, Morton pulled out a tooth for Eben Frost without pain.—The professor now, however, steps in for his share. The Letheon is patented. The surgeons use it in the hospital. Bigelow sends it to Liston, who telegraphs to Edinburgh. Glory! we have conquered pain. The stolen goods are contended for by the first rogue, who finds himself cheated out of the credit by the second, who is a professor, and has the cards over him. They fight over the glittering prize. Meanwhile, the world weeps with joy at the blessed boon, and a thousand thankful hearts throughout the civilized earth send up their grateful prayers to God for the unutterable blessing.

Where is the discoverer—he who thought it first—proved it first—he who ventured all—yea, his life, for the truth—where is Horace Wells?

Defrauded of his honors, betrayed by his friend, deserted by good fortune, his body shattered by the constant use of all sorts of excitants—still experimenting on himself—his mind ill regulated, impulsive, tortured by the cruel fate which seemed to await him; 'twas more than he could bear. Mankind looked here and there eagerly for their benefactor, and found him at last—in the suicide's grave.

We bring before you, reader, in a few words, this mournful story because it is right that we, American physicians, who are proud to claim as ours this greatest gift to medical science, should not neglect to do honor to its real author. His wife lives yet to pray that this may be done. His son asks that his father's claim shall be closely scutinized, and if proved, acknowledged and published to the world. It is substantiated by evidence* too strong to be overthrown—by facts and sworn to by numerous witnesses above suspicion.—Let us then individually examine for ourselves, and then unite with one accord to award tardy justice to the memory of Horace Wells.

---

*See Senator Smith's statement of the question, as laid before the committee of congress, a copy of which, owing to the kindness of a friend, we have before us.

